# Neuro-Ophthalmologic Symptoms Associated With the Moderna mRNA COVID-19 Vaccine: A Case Report

**DOI:** 10.7759/cureus.28523

**Published:** 2022-08-29

**Authors:** Tejas Raxwal, Zeid Akel, Jotsna Naidu, Koravangala Sundaresh

**Affiliations:** 1 Preventive Medicine, Access Health Care Physicians, Zephyrhills, USA; 2 Medical School, Florida State University, Tallahassee, USA; 3 Internal Medicine, HCA Florida Trinity Hospital, Trinity, USA; 4 Infectious Diseases, HCA Florida Trinity Hospital, Trinity, USA

**Keywords:** covid-19, sars-cov-2, mrna covid-19 vaccine, moderna vaccine, optic neuritis, coronavirus

## Abstract

Multiple neuro-ophthalmological symptoms, such as visual field defects, optic neuritis, and eye movement abnormalities, have been reported with coronavirus disease 2019 (COVID-19) infection. It is unknown whether the COVID-19 vaccine can result in similar neuro-ophthalmological symptoms post-vaccination. Here, we describe a case of optic neuritis after the administration of the mRNA COVID-19 Moderna vaccine.

A 47-year-old female presented eight days after receiving the first dose of the Moderna COVID-19 vaccine with impaired vision in the left eye and symptoms consistent with optic neuritis. The patient underwent a workup for infectious etiology, autoimmune diseases, and allergies, which was negative. The patient was treated with a high dose of steroids resulting in the complete resolution of her symptoms. The patient was recommended against the second dose of the mRNA COVID-19 vaccine.

Early detection and treatment of optic neuritis are important to prevent the long-term sequelae of neuritis with impaired vision.

## Introduction

In 2019, a severe acute respiratory syndrome (SARS-CoV-2) causing pulmonary infection was reported in Wuhan city in China, which was termed coronavirus disease 2019 (COVID-19) [1]. Since then, the infection has evolved into a pandemic, and at the time of this writing, the pandemic is still ongoing.

Numerous COVID-19 vaccines have been developed and are in various stages of development. In the United States, two COVID-19 mRNA vaccines by Pfizer-BioNTech and Moderna have been approved for the prevention of COVID-19 [2], in addition to the Johnson and Johnson vaccine.

COVID-19 infection has manifested with multiple neuro-ophthalmological symptoms, such as optic neuritis, visual field defects, and eye movement abnormalities [3]. It is unclear whether patients may have a possible manifestation of neuro-ophthalmological symptoms post-COVID-19 vaccination. Data regarding optic neuritis post-COVID-19 vaccination are limited.

## Case presentation

A 47-year-old female healthcare worker presented to the hospital with impaired vision in her left eye. She could not see letters on the computer with her left eye due to blurred vision. Her vision in the right eye was normal. The patient complained of discomfort in the left eyebrow and on the left side of the head. She reported shooting pain between the left ear and in the left eye. The patient noted a gritty sensation around her left eye. She denied any fever, chills, dyspnea, or cough. She did not complain of dizziness, diplopia, or dysphagia. She had no focal neurodeficit or dysarthria. She was alert and oriented to place, time, and person. She had a medical history of well-controlled hypertension treated with amlodipine for one year. She complained of persistent fatigue since she received the first dose of the Moderna vaccine eight days prior to the onset of her visual symptoms. The patient had no prior history of impaired vision or autoimmune disorder.

According to the institutional stroke protocol, she underwent computed tomography (CT) of the head and a CT angiogram of the head and neck, which were negative. She underwent magnetic resonance imaging (MRI) of the head, C spine, and optic nerve, which was also normal.

The ophthalmology examination showed normal external examination with no signs of ptosis. The conjunctiva and sclera were normal. The corneas were clear and transparent. The periorbital area was normal. Her extraocular muscle movements were intact bilaterally without pain in any direction. A left afferent pupillary defect (APD) was noted. The pupils were 2 mm round and reactive with positive APD in the left eye. Visual acuity was a Jaeger score of 1 (Snellen 20/25) in the right eye and a Jaeger score of 5 (Snellen 20/50) in the left eye. Confrontation visual field testing was normal. Anterior segment examination was unremarkable with intraocular pressure by Tono-pen of 15 in the right and 16 in the left eye.

Dilated funduscopic examination showed trace nuclear sclerosis of the lens. The vitreous was clear bilaterally, and no macular abnormalities were noted. No evidence of retinal detachment was noted. Vessels were normal with no signs of intraretinal hemorrhage. The optic nerve showed a cup-to-disk ratio of 0.3. There was no pallor of the optic nerve and no evidence of papilledema bilaterally.

Optic neuritis can result from inflammatory, autoimmune, or infectious disorders. To evaluate for inflammatory disorders, laboratory workup including complete blood count, complete metabolic profile, coagulation profile, erythrocyte sedimentation rate, C-reactive protein, and D-dimer were tested, which were normal. She was tested for COVID-19 infection, which was negative. The patient was also evaluated for COVID-19 antibody, which was negative. Serum Lyme titer, *Bartonella* serology panel, hepatitis profile, and human immunodeficiency virus serology screen were negative, ruling out the infectious etiology of optic neuritis. Vitamin B12, folic acid, and thyroid-stimulating hormone were normal. Homocysteine, anti-phospholipid antibody, lupus anticoagulant, protein C, protein S, antithrombin III, serum protein electrophoresis, anti-nuclear antibody (ANA), rheumatoid arthritis (RA) factor, perinuclear anti-neutrophil cytoplasmic antibodies (P-ANCA), cytoplasmic anti-neutrophil cytoplasmic antibodies (C-ANCA), and factor V Leiden factor were negative, ruling out an autoimmune etiology. An electrocardiogram was normal. An echocardiogram showed a normal ejection fraction with mild pulmonary hypertension with systolic pulmonary artery pressure of 45 mmHg.

Clinically, a diagnosis of optic neuritis was made. She was administered a high dose of methylprednisolone of 1,000 mg daily for two days, followed by prednisone 1 mg/kg (60 mg daily) for a 14-day course with taper for the last five days. She reported remarkable improvement in her vision the following day. The pain in the left side of the face and head persisted, there were no signs of Bell’s Palsy, but a clinical possibility of cranial nerve VII involvement was suspected.

She was subsequently readmitted to the hospital one week later with new complaints of severe weakness and dyspnea. CT pulmonary angiogram was negative for pulmonary embolism. Her fatigue was thought to be due to steroids, but given the risks versus benefits of optic neuritis treatment, she was recommended to undergo the treatment dose as prescribed. She was recommended an MRI of the brain to evaluate for multiple sclerosis in six months.

The patient had an outpatient follow-up appointment with ophthalmology with a normal eye examination and normal vision. The patient was evaluated by an allergist and rheumatologist to exclude an autoimmune disorder as an etiology for optic neuritis. Her autoimmune workup was negative, including ANA, RA factor, P-ANCA, and C-ANCA. The patient was recommended against receiving the second dose of the mRNA COVID-19 vaccine.

## Discussion

Adverse reactions to vaccines are usually rare and often attributed to various vaccine components [4]. At present, the etiology of adverse reactions to Moderna mRNA vaccines is not clear. The Centers for Disease Control and Prevention (CDC) recommends avoiding both mRNA COVID-19 vaccines in individuals with a history of anaphylaxis to polyethylene glycol (PEG), PEG derivatives, or polysorbate. Recently, the CDC has reported myocarditis following the mRNA vaccine, and various reports of myocarditis in children and adults have been published; however, the occurrence of myocarditis in response to a COVID-19 vaccine is statistically less than the occurrence of myocarditis in response to a COVID-19 infection in both adults and children.

In COVID-19 infections, symptoms of optic neuritis, diplopia, and ptosis have been described. Eye movement abnormalities, nystagmus, and visual field defects have been reported as well. In a study examining the neurological complications of COVID-19, one case of optic neuritis was reported during the recovery phase [5]. We report a patient who developed neuro-ophthalmic symptoms following the mRNA Moderna COVID-19 vaccine.

Other studies have reported similar neuro-ophthalmologic complications following other COVID-19 vaccines Ad26.COV2.S vaccine (Janssen Pharmaceutical Companies) [6], Pfizer-BioNTech vaccine, AstraZeneca-Oxford vaccine [7], Covishield vaccine [8], and Sinopharm vaccine [9]. Similar neuro-ophthalmologic complications have been reported following the administration of the measles vaccine [10,11].

Optic neuritis is an inflammatory condition that causes acute monocular visual loss most likely due to optic nerve demyelination. A possible mechanism for the demyelination in optic neuritis is immune-mediated, but the specific mechanism and target antigens remain unknown. However, possible mechanisms could be due to an inflammatory response to a specific monoclonal antigen, a post-viral inflammatory syndrome, or sequelae of a pro-inflammatory state. Moreover, systemic abnormalities such as hypoxia and hypercoagulability may play a role.

Early diagnosis and treatment are essential to prevent long-term sequelae. High-dose steroid treatment should be considered in selected patients with optic neuritis as there is some evidence that this treatment may delay the onset of multiple sclerosis and hasten visual recovery. Alternative treatments for acute neuroimmunological diseases include intravenous immunoglobulin and plasma exchange. Further, treatment with immunomodulatory therapies, a so-called disease-modifying therapy for patients with optic neuritis and abnormal brain MRIs, may be considered in select patients.

In patients with a pre-existing condition of autoimmune disorders, careful screening and risk stratification should be considered in conjunction with an allergist and immunologist before vaccination [12]. Every patient should be carefully evaluated for a possible allergic reaction before denying vaccination in view of the potentially life-saving benefit of vaccination in the setting of a global pandemic.

## Conclusions

Neuro-ophthalmologic complications can manifest following the COVID-19 mRNA vaccine Moderna. Optic neuritis, an inflammatory, demyelinating condition that manifests with acute, usually monocular visual loss, may be complicated after COVID-19 vaccination. Early detection and treatment of optic neuritis are vital to prevent the long-term sequelae of optic neuritis with impaired vision. High-dose steroids may prevent the long-term consequences of neuritis. Patients with complications of optic neuritis might benefit from clinician recommendations against subsequent vaccine doses.
